# Genital Evolution: Why Are Females Still Understudied?

**DOI:** 10.1371/journal.pbio.1001851

**Published:** 2014-05-06

**Authors:** Malin Ah-King, Andrew B. Barron, Marie E. Herberstein

**Affiliations:** 1 Centre for Gender Research, Uppsala University, Uppsala, Sweden; 2 Centre for Gender and Future Research, Marburg University, Marburg, Germany; 3 Department of Biological Sciences, Macquarie University, Sydney, Australia

## Abstract

The diversity, variability, and apparent rapid evolution of animal genitalia are a vivid focus of research in evolutionary biology, and studies exploring genitalia have dramatically increased over the past decade. These studies, however, exhibit a strong male bias, which has worsened since 2000, despite the fact that this bias has been explicitly pointed out in the past. Early critics argued that previous investigators too often considered only males and their genitalia, while overlooking female genitalia or physiology. Our analysis of the literature shows that overall this male bias has worsened with time. The degree of bias is not consistent between subdisciplines: studies of the lock-and-key hypothesis have been the most male focused, while studies of cryptic female choice usually consider both sexes. The degree of bias also differed across taxonomic groups, but did not associate with the ease of study of male and female genital characteristics. We argue that the persisting male bias in this field cannot solely be explained by anatomical sex differences influencing accessibility. Rather the bias reflects enduring assumptions about the dominant role of males in sex, and invariant female genitalia. New research highlights how rapidly female genital traits can evolve, and how complex coevolutionary dynamics between males and females can shape genital structures. We argue that understanding genital evolution is hampered by an outdated single-sex bias.

## Abstract 2

In many animal groups genital structures appear to have evolved extremely rapidly, prompting enduring interest in why this is so. Throughout this literature there remains a bias towards studying male genitalia; here we examine the extent of that bias and its possible causes.

The tremendous diversity of male genitalia has been described as one of evolutionary biology's greatest enigmas [1], and several hypotheses have been put forward to explain this diversity (Box 1). Hypotheses explaining this diversity include the lock-and-key hypothesis of species isolation (in which male “keys” fit species-specific female “locks”) [2], the pleiotropy hypothesis suggesting that genital morphology is due to pleiotropic effects of natural selection on other traits [3], female choice [4], sperm competition (competition between sperm from different males for an egg) [5], and sexual conflict (occurring when the two sexes have different optimal fitness strategies for reproduction potentially leading to evolutionary arms races between males and females) [6],[7]. An influential 2004 review by Hosken and Stockley [1] concluded that the field has arrived at a general consensus that sexual selection plays an important role in the evolution of genitalia. A response to Hosken and Stockley [1] by Méndez and Córdoba-Aguilar [8] highlighted the then well-known problem that the field of genital evolution as a whole paid far too little attention to the role of females in sexual dynamics, arguing that most studies measured male genital traits and ignored females, hence overlooking the intricate dynamics between the form and function of genitals. However, just as Darwin predicted reciprocal evolution of pollinator proboscis length and floral tube length [9],[10], genitalia in internally fertilizing species ought to involve an evolutionary dynamic between both sexes.

**Box 1. Hypotheses proposed to explain selective forces acting on genital evolution**

**Lock-and-key**: Genital divergence is selected for by avoidance of hybridization, where only individuals with matching genitalia can successfully mate. It was originally proposed by Dufour [2] and has enjoyed enduring consent from taxonomists.

**Pleiotropy** (neutral evolution): Genitalia evolve indirectly via selection of other traits that are genetically correlated and thus variation in genitalia is selectively neutral [3].

**Sexual selection**: Genital shape and size is under selection through differential fertilization success. Three mechanisms of sexual selection are most frequently evoked: 1) cryptic female choice [4], where male genital shape affects female manipulation of sperm; 2) sexual conflict [39], where genital morphology result from a conflict over fertilization control between males and females; and 3) sperm competition, where variation in male genitalia influences fertilization success through sperm placement or displacement, e.g., [5].

Despite this obvious statement, variation in male genitalia is most often discussed without consideration of their female counterparts. It has been repeatedly noted that a lack of study of female genitalia has seriously hampered comprehension of genital evolution [8],[11],[12]. The lock-and-key hypothesis (see Box 1) has been largely dismissed due to lack of variation among females, and hence an apparent lack of a species-specific “lock” for the “key” [4],[13]. However, taxonomic data on female genitalia are scarce, which may explain why studies that have relied on such data have failed to find evidence for coevolution of male and female genitalia [12].

Males are unlikely to have complete control over sperm usage in internally fertilizing species, and females are expected to influence fertilization and hence paternity. It is therefore appropriate to consider the effects of female genitalia alongside those of the male [8],[12]. Given that the field has greatly expanded since the publication of Hosken and Stockley's review [1], we examined whether the initially observed male bias has changed by analyzing the number of studies that have investigated the evolution of male genitalia, female genitalia, or that of both sexes in each year from 1989 to 2013 (Box 2).

**Box 2. Literature analysis methods**

We broadly classify genitalia as male and female structures that physically interact during sperm transfer, but recognize that many studies do not define genitalia and may include additional reproductive structures under that term. However, since we are concerned about how the field of genital evolution treats male and female structures, we accepted the authors' definition of genitalia.

We also recognize that there are many mostly morphological papers that describe male and female reproductive structures, including genitalia. While these studies may include valuable information on how genitalia function during copulation, we only included them if they addressed questions about the evolution of genitalia.

We searched the Web of Science (May 14, 2013) for studies on genital evolution using the following search terms: “sexual selection & genital*,” “evolution & genital*,” and “speciation & genital*.” This search resulted in over 2,200 hits with some overlap between the search terms.

Because some female genital structures may be overlooked by these search terms, we performed additional searches in the Web of Science with the following terms: spermatheca AND Topic = (sexual selection or evolution or speciation) NOT Topic = (genital*); vagina AND Topic = (sexual selection OR evolution OR speciation) NOT Topic = (genital*); bursa AND Topic = (sexual selection OR evolution OR speciation) NOT Topic = (genital*); reproductive tract AND Topic = (sexual selection OR evolution OR speciation) NOT Topic = (genital*). These additional searches generated ∼900 hits. After eliminating overlap from the different searches, we filtered the ∼3,000 papers (based on titles and abstracts) to eliminate any that did not consider an investigation of genital evolution. Many papers returned by our search terms were purely descriptive/morphological or otherwise unrelated to our focal question. This generated a library of 646 papers. We investigated each of the 646 papers, excluding pure descriptions, reviews, opinion papers, replies, studies that did not investigate genital variation, and a small number of papers (eight) that were unavailable in full text. A small number of papers (seven) that measured female reproductive tract traits in conjunction with male sperm traits were also excluded, as these studies did not consider genitalia *per se*. This reduced the list to 364 papers for which we noted the year of publication; the broad taxonomic group, genus, and species name; whether the male genitalia, the female genitalia, or both were investigated; and the selective mechanism the authors attributed to the observed structure and/or variation in genitalia.

Some authors attributed the observed genital structures to sexual selection broadly, without specifying a particular mechanism (e.g., sperm competition, cryptic female choice, or sexual conflict). These studies were coded as “sexual selection.” Others attributed multiple mechanisms including sexual selection and others, such as lock-and-key, pleiotropy, or natural selection. These were coded as “multiple.” Studies that did not offer an explanatory mechanism were coded as “none.” Studies on speciation and hybrid zones did not always use the term “lock & key” explicitly, but implied reproductive isolation via genital morphology and hence we coded them under “lock & key” (see Table S1). In our data summary (Figure 1, Figure 2, Figure 3) we only included categories (taxonomic group or mechanism) with more than five studies. This excluded nine taxonomic groups and two evolutionary mechanisms (pleiotropy and natural selection).

**Figure 1:**
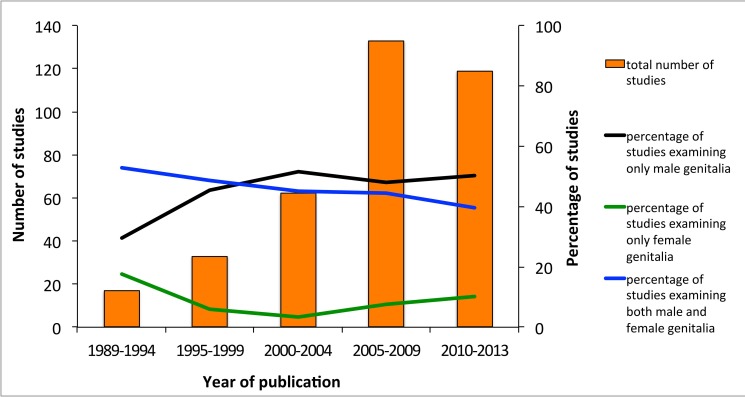
Publication trends: focus on male, female, or both sexes. The number of published papers on animal genitalia analyzed from 1989 to the present, and the percentage of studies that examine only male genitalia, only female genitalia, and both male and female genitalia.

**Figure 2:**
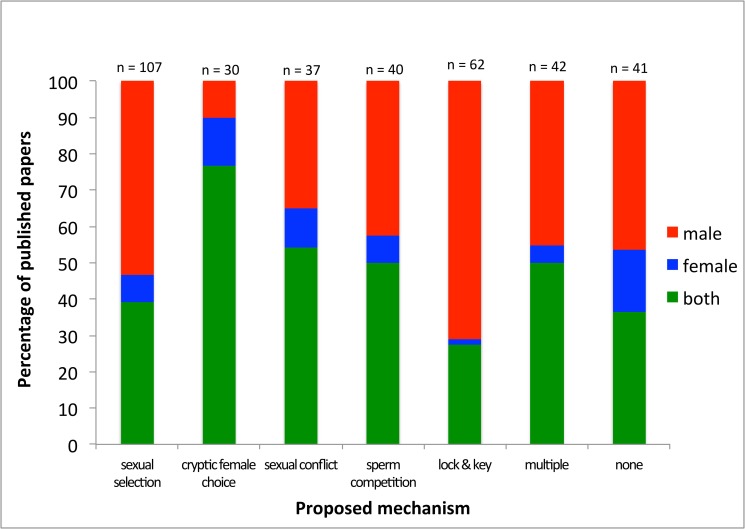
Publications by mechanism. The percentage of published papers that investigated male genitalia, female genitalia, or both against the proposed mechanisms as suggested by the respective authors.

**Figure 3:**
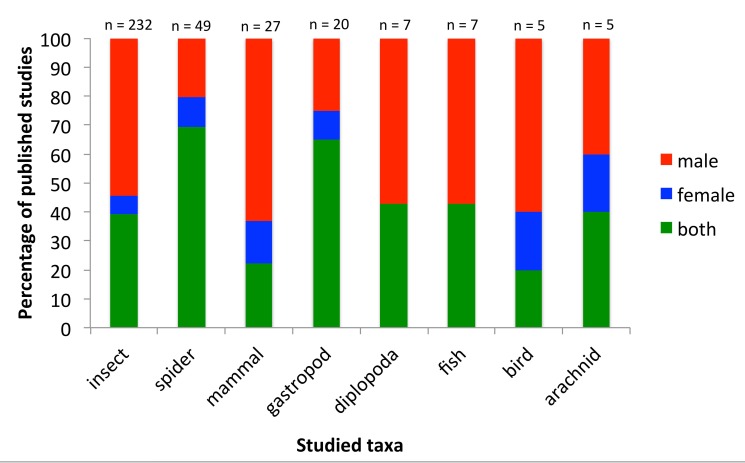
Publications by taxonomic group. The percentage of published papers analyzed that investigated male genitalia, female genitalia, or both against the focal taxonomic group.

We found that the topic of genital evolution has enjoyed a dramatic increase in publication numbers, rising from under five studies per year in the early 90s to over 40 studies in 2012 alone. The most dramatic increase occurred after the 2000–2004 period, coinciding with Hosken and Stockley's [1] review (Figure 1).

While the field has grown, studies on the evolution of animal genitalia remain dominated by investigations of males. Of the 364 studies we analyzed, 48.6% (177) were on male genitalia, only 7.7% (28) on female genitalia, and 43.7% (159) on both male and female genitalia. There seems to have been an even stronger single-sex bias toward male-only studies from 2000 onwards (Figure 1) despite a clearly articulated call for greater consideration of the roles of females by Méndez and Córdoba-Aguilar in 2004 [8]. This bias is seen in most subdisciplines of genital evolution research regardless of whether studies were exploring speciation or aspects of sexual selection (Figure 2). A notable, and perhaps not surprising, exception is studies of cryptic female choice, where single-sex studies constitute only around 24% of the publications. Publications exploring the lock-and-key hypothesis show the greatest bias with over 70% of studies only considering male genitalia. The majority of publications on animal genitalia are based on insects, with spiders as the second most common model. The taxonomic groups that suffered the least single-sex bias were spiders and gastropods, whereas mammals showed the greatest bias (Figure 3).

## Why a Male Bias in Studies?

Why is there still a consistent male bias in genital studies of internally fertilizing species? It has been proposed that this bias arises because male genitals are often more rigid and easier to study than female organs [11]. This may indeed have hampered the investigation of female genitals [11], but we question whether this explanation is the sole reason for the pattern of male bias uncovered here, since the bias differs strikingly between different mechanisms investigated (Figure 2). In many insects the internal genitalia may be soft and membraneous, but given the availability of modern techniques this should not limit research investigations if the investigators are interested in the question. For example, micro-computed tomography (CT) scanning of millipede genitalia has revealed high complexity in female genitalia as well as large mechanical correspondence between male and female genitalia in copula [14]. It may be that female genitalia with obvious and quite variable external elements encourage further investigation of internal components. This may explain the high proportion of studies on spiders that considered the interaction of male and female genitalia (Figure 3).

An alternative explanation for the observed male bias is that female genitalia might not vary much, justifying the focus on variation in male organs. Nevertheless, several detailed studies show significant inter- and intraspecific variation in female genitalia [13],[15]–[18]. For example, spiders show a large diversity in female genitalia among entelegyne spiders and the form of female genitals is very often species-specific [19]. Waterfowl demonstrate extreme variation in vaginal morphology, with some species such as the mallard *Anas platyrhynchos* having a highly elaborate and convoluted vagina [20]. Other examples include primate genitalia that show interspecific variation in sexual signalling (e.g., [21]). Interspecific differences and rapid evolution of female genitalia in sepsid and drosophilid flies [18],[22] suggest that variation in the female tract can contribute to reproductive isolation. Female genitalia can even be polymorphic within species [15], clearly showing that female genital morphology is rapidly evolving and subject to active selective forces.

Since it was first proposed, sexual selection theory has been influenced by cultural assumptions about males and females, such as Darwin's initial proposal of females being generally “coy” [23]. Although the mechanism of male-male competition was readily accepted by Darwin's contemporary biologists, female choice was questioned as investigators thought it uncertain whether females had the mental abilities for executing mate choice [24]. In time and due to criticism from female perspectives, investigators have abandoned gender stereotypes, such as females being generally passive [25]. However, males, their characteristics, and behavior have often been the first object of investigation in the field, later followed by questions about females and their characteristics. For example, sperm competition (male-male competition on the gamete level) was studied a long time before the corresponding idea of cryptic female choice was suggested, and at the time, the latter was generally thought barely credible [26]. Since Eberhard's [27] in-depth treatment of cryptic female choice, researchers have paid more attention to female genitalia since the possibility of cryptic choice forced a more careful analysis of the possible roles of females [1]. Hence, male bias has historically influenced the assumptions and questions pursued in sexual selection research. Even today the dominant paradigm in the field holds assumptions that steer researchers toward focusing on male subjects more than females: of higher male variance in reproductive success, of males gaining more by multiple mating than females, and of females being choosier and less eager than males [28]. Indeed, the theoretical assumption that male components of sexual selection are more important than female ones may be one explanation for the biased focus on male genitals in the field [11]. The generality of these assumptions is now being questioned and reevaluated [29]–[31].

Another source of bias may be the gender of the investigators themselves, e.g., are women more prone to investigate variation in females and vice versa? We partitioned the papers according to the gender of the principal investigator and calculated the percentage of papers focusing on female, male, or both subjects (Table 1). The table shows that the male bias in studies is equally distributed among female and male authors, hence we conclude that the gender of the investigators cannot account for the observed bias.

**Table 1:** Comparison between female and male principal investigators' focus on sex of subjects.

	Sex of subjects			
Principal investigator	Female	Male	Both	Sum
**Female**	7 (7.8%)	46 (51.1%)	37 (41.1%)	90
**Male**	21 (8.0%)	121 (46.3%)	119 (55.5%)	261

Numbers of papers (and percentage) of studies that focused on females, males, or both sexes authored by female/male principal investigators. The male bias is equally distributed among female and male authors. Thirteen studies were excluded because we were unable to determine the author's gender.

Given the difference in bias among different research areas (Figure 2), we suggest that certain research questions steer researchers toward focusing more on males and thus to overlooking female features. Furthermore, research on different animal groups differs in focus on males/females (Figure 3), which may both be an effect of the sex differences in ease of studying genitalia (e.g., both male and female spiders have external genitalia) and the kind of questions that are pursued in that specific group. We conclude that there seems to be no biological justification for why female genitalia are understudied, and suggest that the bias reflects now outdated assumptions about the unimportance of, or lack of, variation in female genitalia in sexual evolutionary dynamics.

## The Importance of Studying Female Genitalia

While experimental studies exploring how evolutionary forces have shaped female genital evolution are still a minority of the total genital evolution literature, they provide a disproportionately significant insight into evolutionary sexual dynamics.

Rapid evolution of female genitalia can be driven by sexual conflict, as a response to male wounding of females during copulation. Comparative analysis of the *Drosophila melanogaster* species subgroup revealed the rapid evolution of paired pocket-like structures in the female genital tract. These have evolved to accommodate the equally rapidly evolving paired spines on the male genitalia, and prevent wounding of the female by these spines during copulation [32]. Similarly, in seed beetles males with larger spines on their penises cause more damage to females during mating. Phylogenetic analysis of the group shows that females have evolved a counter-adaptation to the spines by increasing the amount of structural connective tissue in their genital tract, thereby reducing the extent of wounding by males [33]. Studies such as these demonstrate that the functions of male armaments are difficult to interpret without exploring the impact on females and their evolutionary counter-adaptations.

Sperm competition depends on the coincident occurrence of sperm from more than one male in the female. There have been numerous studies on the specialized male penile structures that remove competitor sperm from the female. Too often the female is assumed to be an invariant container within which all this presumed scooping, hooking, and plunging occurs [34]. For example, the male virga of the earwig *Euborellia plebeja* is considered a classic sperm competition adaptation. It is as long as the male body, and possesses an apical fringe of hairs. As the virga is inserted and removed prior to ejaculation, a reasonable assumption is that its structure enables efficient allosperm removal [35]. However, when examining the female genitalia, a different story emerges [36]. The sperm storage organs of the female are longer than the virga of the male, which prevents later mates from effectively removing the sperm of prior males. Hence, the morphology of the spermatheca limits sperm displacement and enables female control of sperm retention [36].

**Figure 4:**
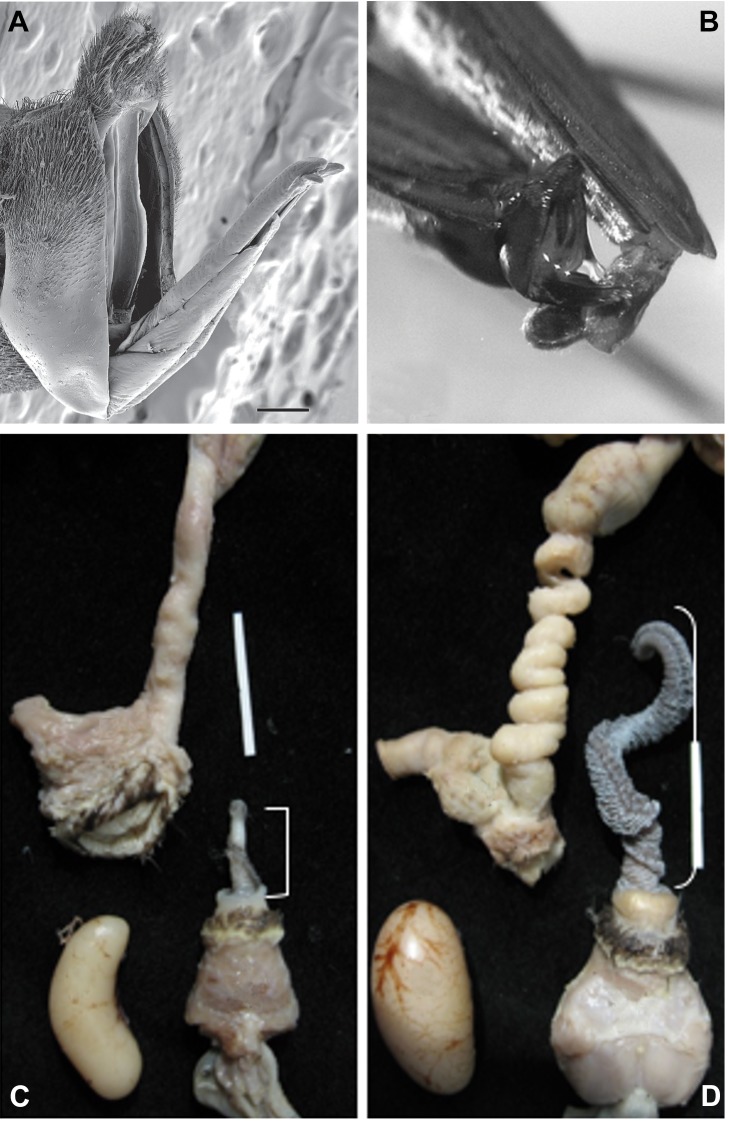
Examples of studies investigating the evolution of both sexes' genitalia. The figure shows examples of significant studies investigating both male and female genitalia. (A) The mobile female genitalia in the water strider *Gerris gracilicornis* with a genital shield that can block forced copulations, and the interlocking of female and male genitalia (B). Figure republished from Han and Jablonski (2009) [37] under a CC-BY license. (C) and (D) show the covariation of female and male genitalia in different species of ducks in which the level of forced copulation covaries with length of the phallus and elaborateness of vaginas. (C) Harlequin duck (*Histrionicus histrionicus*) with short phallus, no forced copulations, and simple vaginas and (D) long-tailed duck (*Clangula hyemalis*) with high levels of forced copulation, long phallus, and elaborate vaginas (size bars = 2 cm). Figure republished from Brennan et al. (2007) [20] with CC-BY license.

Changes in female genitalia have also driven changes in male courtship behavior. In water striders *Gerris gracilicornis* females have evolved a genital shield, which seems to effectively block forced copulations by males (Figure 4). As a consequence males have evolved new forms of courtship behavior to advertize to females and attract mating opportunities rather than coerce [37]; but should that fail, males will lure potential predators into the area to presumably intimidate a female into accepting a coercive mating attempt [38].

In waterfowl elaborate vaginal morphology (which can involve complex convolutions and several dead-end sacs) has coevolved with male phallus length, which itself coevolved with the frequency of apparently forced extra-pair copulations [20] (Figure 4). These vaginal adaptations may have evolved as an anatomical mechanism of cryptic female choice in species in which forced attempted copulations are common to provide an anatomical mechanism to block or limit penetration of the phallus in forced copulation [20].

These studies illustrate the extremely rich evolutionary dynamics that are revealed when the role of female genitalia is considered alongside that of the male. Studies addressing only one sex are at risk of examining just one side of a very complex equation and may be more prone to misinterpreting the highly complex coevolutionary dynamic that can occur between the sexes. In contrast, studies that consider the coevolution of male and female genitalia have proved highly influential for our understanding of the function and evolution of animal genitalia.

## Supporting Information

Table S1 Database of papers considered in this analysis.
(XLSX)

## Abbreviations

CT: computed tomography
